# Role of Nrf2 and Its Activators in Respiratory Diseases

**DOI:** 10.1155/2019/7090534

**Published:** 2019-01-08

**Authors:** Qinmei Liu, Yun Gao, Xinxin Ci

**Affiliations:** ^1^Institute of Translational Medicine, The First Hospital of Jilin University, Changchun 130001, China; ^2^Department of Respiratory Medicine, The First Hospital of Jilin University, Changchun 130001, China

## Abstract

Transcription factor nuclear factor erythroid 2-related factor 2 (Nrf2) is a major regulator of antioxidant response element- (ARE-) driven cytoprotective protein expression. The activation of Nrf2 signaling plays an essential role in preventing cells and tissues from injury induced by oxidative stress. Under the unstressed conditions, natural inhibitor of Nrf2, Kelch-like ECH-associated protein 1 (Keap1), traps Nrf2 in the cytoplasm and promotes the degradation of Nrf2 by the 26S proteasome. Nevertheless, stresses including highly oxidative microenvironments, impair the ability of Keap1 to target Nrf2 for ubiquitination and degradation, and induce newly synthesized Nrf2 to translocate to the nucleus to bind with ARE. Due to constant exposure to external environments, including diverse pollutants and other oxidants, the redox balance maintained by Nrf2 is fairly important to the airways. To date, researchers have discovered that Nrf2 deletion results in high susceptibility and severity of insults in various models of respiratory diseases, including bronchopulmonary dysplasia (BPD), respiratory infections, acute respiratory distress syndrome (ARDS), chronic obstructive pulmonary disease (COPD), asthma, idiopathic pulmonary fibrosis (IPF), and lung cancer. Conversely, Nrf2 activation confers protective effects on these lung disorders. In the present review, we summarize Nrf2 involvement in the pathogenesis of the above respiratory diseases that have been identified by experimental models and human studies and describe the protective effects of Nrf2 inducers on these diseases.

## 1. Oxidative Stress and Antioxidant Responses in Respiratory Diseases

In the past few decades, environmental issues due to manmade and natural factors have sharply increased the incidence of malignant and nonmalignant respiratory diseases. Therefore, the reason underlying why the respiratory system is so easily affected by environmental problems and the pathogenesis of respiratory diseases has attracted increasing attention.

As the location of gas exchange, the airways with large surface area constantly interface with the external environment and are exposed to various airborne toxicants especially inhaled oxidants (e.g., environmental ozone, particles, and cigarette smoke) [1]. Due to the special characteristics of anatomy and physiology, the airways are placed in highly oxidative microenvironments. Therefore, redox homeostasis in the airways can be easily disturbed, which is referred to as oxidative stress [2]. Oxidative stress is a common status defined as the imbalance between reactive oxygen species (ROS) production and antioxidant capacity in cells under temporary or constant stimulation of the abundant oxidant stressors [3]. Recently, oxidative stress has been proven to be associated with the pathogenesis of diverse acute and chronic respiratory diseases, including respiratory infections, acute respiratory distress syndrome (ARDS), chronic obstructive pulmonary disease (COPD), asthma, idiopathic pulmonary fibrosis (IPF), and lung cancer [4–6]. In the lungs of individuals with these diseases, the disruption of redox balance is always observed and may be represented by increased biomarkers of oxidative stress.

Nevertheless, during the long journey of life evolution, the organism has developed a series of antioxidant responses to counteract the toxicity of oxidative stress. The antioxidant system in cellular response includes either proteins (e.g., enzymes) or small molecules (e.g., vitamins C and E). As enzymes have been proven to play a significant role in life cycles, their effects on antioxidant defense have been investigated extensively. Direct antioxidant enzymes refer to classical enzymes including superoxide dismutases (SODs), catalase, and glutathione peroxidase (GPx), while indirect antioxidant enzymes mainly refer to phase 2 detoxifying enzymes such as glutathione-S-transferase (GST) isozymes, catalytic and modifier subunits of *γ*-glutamyl cysteine ligase (GCLC, GCLM), and NADP(H):quinone oxidoreductase (NQO1). Moreover, the stress response protein heme oxygenase (HO-1) is reported to be a particularly potent antioxidant protein [7–9]. Antioxidant substances are present in relative abundance in both epithelial lining fluid (ELF) and lung tissues, as airways are places where detoxification reactions routinely occur, and protect the lungs from oxidative insults in healthy individuals [2]. Unfortunately, in susceptible individuals, the depletion of GSH and other antioxidants can occur, and these individuals are prone to developing oxidative respiratory diseases. Moreover, the protective role of the antioxidant system in the prevention of these respiratory diseases may also be proven by several therapies aimed at defending against oxidative stress that have already been applied in BPD, COPD, and IPF, such as treatment with vitamins C and E or N-acetylcysteine (NAC, a GSH precursor) or treatment with polyethylene glycol-conjugated SOD and catalase.

## 2. Nrf2-Mediated Antioxidant Pathway and Respiratory Diseases

Although the functional mechanisms are diverse in the antioxidant system, a large number of typical phase 2 detoxifying enzymes and the stress response protein HO-1 are regulated by the transcription factor nuclear factor erythroid 2-related factor 2 (Nrf2), which indicates that this transcription factor is a possible and imperative upstream regulator of antioxidative responses that maintains cellular redox homeostasis and reduces severe oxidative damage [10–13].

Nrf2, which belongs to the cap “n” collar (CNC) family of transcription factors, is a major transcription factor that counteracts oxidative stress and inflammation through the coordinated induction of antioxidant response element- (ARE-) driven cytoprotective gene transcription [14, 15]. The classical mechanisms of Nrf2 activation include oxidative modification and conformational changes in its major repressor protein Kelch-like ECH associated protein 1 (Keap1), followed by Nrf2 stabilization due to escape from ubiquitination by Cul3-Rbx1. This molecular model is proven by the constitutive accumulation of Nrf2 in the nuclei of Keap1-knockout mice [16]. In fact, Nrf2 consists of six functional domains recognized as Nrf2-ECH homologies 1–6 (Neh1–6), including the Keap1 binding domain (Neh2) and the leucine zipper domain (Neh1), through which Nrf2 can heterodimerize with small Maf or Jun proteins and then bind to ARE [17]. The Nrf2 repressor keap1 is a cytoplasmic and cysteine-rich protein whose N-terminal BTB domain binds to Cullin 3- (Cul3-) Rbx1, while the C-terminal DGR domain binds to Nrf2. Keap1 represses Nrf2 by serving as a substrate adaptor for the Cul3-containing E3 ubiquitin ligase complex. Under physiological conditions, Keap1 holds Nrf2 in the cytoplasm and ubiquitinates Nrf2 to facilitate its degradation by the 26S proteasome. However, when oxidative stimuli exist, the cysteine residues of Keap1 can be modified, leading to Nrf2 stabilization and accumulation in the nucleus [18]. Several mechanisms for the activation of Nrf2 by Keap1 have been proposed as the following up to now [17, 19, 20]. (1) Keap1 dissociation: the modification of a cysteine in Keap1 makes Nrf2 dissociate from Keap1. (2) Keap1 hinge and latch: as a more extensively accepted model, Nrf2 binds with the Keap1 homodimer through a high-affinity ETGE motif as the “hinge” and a low-affinity DLG motif as the “latch.” The modification of cysteine in Keap1 leads to a conformational change but does not trigger the dissociation of Nrf2, which may inhibit ubiquitin binding onto Nrf2 by disrupting the weak latch binding site. (3) Keap1 ubiquitination: the modification of a cysteine in Keap1 moves the ubiquitin conjugation from Nrf2 to itself. Nevertheless, the precise molecular mechanisms behind how Nrf2 bypasses the Keap1 gate under stressed conditions still remain to be elucidated. Several studies in recent years have proposed fairly intriguing theories. For example, Cys-151 in the BTB domain of Keap1 is likely to play an important role in response to Nrf2 activators, and this action may be associated with its destructive effect on the interaction of Keap1 with Cul3 [21]. Diverse Nrf2 activators activate Nrf2 signaling through this canonical mechanism, and the mutation of cysteine 151 to serine (Keap1-C151S) in Keap1 completely abolishes the Nrf2 upregulation [22, 23]. Other studies indicate that Cys-273 and Cys-288 in Keap1 may also contribute to the structural integrity and activity of Keap1 for maintaining ubiquitin ligase activity [24]. In addition, there are also Keap1-independent pathways for Nrf2 activation, among which protein kinases play an essential role. A previous study showed that phosphorylation at a specific amino acid residue of Nrf2 can increase its stability and transactivation activity [8]. Typical protein kinase pathways include phosphatidylinositol 3-kinase (PI3K), MAPKs, PKC, and glycogen synthase kinase-3 (GSK-3). The phosphorylation of Nrf2 by PI3K, PKC, c-Jun, N-terminal kinase (JNK) and extracellular signal-regulated protein kinase (ERK) confers positive regulation, whereas p38 MAPK regulates the Nrf2 pathway both positively and negatively [19, 25–29]. Recently, a noncanonical pathway of Nrf2 activation involving autophagy has attracted increasing attention due to its double effect. This pathway is closely associated with the autophagy substrate protein sequestosome 1 (SQSTM1 or p62). p62 can compete with Nrf2 for Keap1 binding, sequester Keap1 into the autophagosome, and allow Nrf2 stabilization and accumulation [30]. However, autophagy-mediated Nrf2 activation has both positive and negative effects: induction of autophagy leads to sequestration of Keap1-p62 complexes into autophagosomes and lysosomal-mediated degradation of Keap1, resulting in controlled Nrf2 activation, and exerts protective effects, while autophagy dysregulation results in prolonged Nrf2 activation in a pathological state and exerts detrimental effects.

Moreover, recent studies have shown that in addition to Keap1, other pathways involving cullin adaptor proteins also direct the ubiquitination of Nrf2. For instance, the phosphorylation of Nrf2 at specific serine residues in the Neh6 domain by GSK-3 forms a degradation part for recognition by the ubiquitin ligase adapter Skp1-Cul1-F-box protein (SCF)/*β*-TrCP (*β*-transducin repeat-containing protein) and proteasome degradation by the Cullin1/Rbx1 complex [31, 32]. Similarly, WDR23, a substrate receptor for the Cullin4- (CUL4-) DDB1 (damaged DNA-binding protein 1) E3-ubiquitin ligase, binds with the Neh2 domain of Nrf2 and negatively regulates the level and activity of Nrf2 [33].

**Figure 1:**
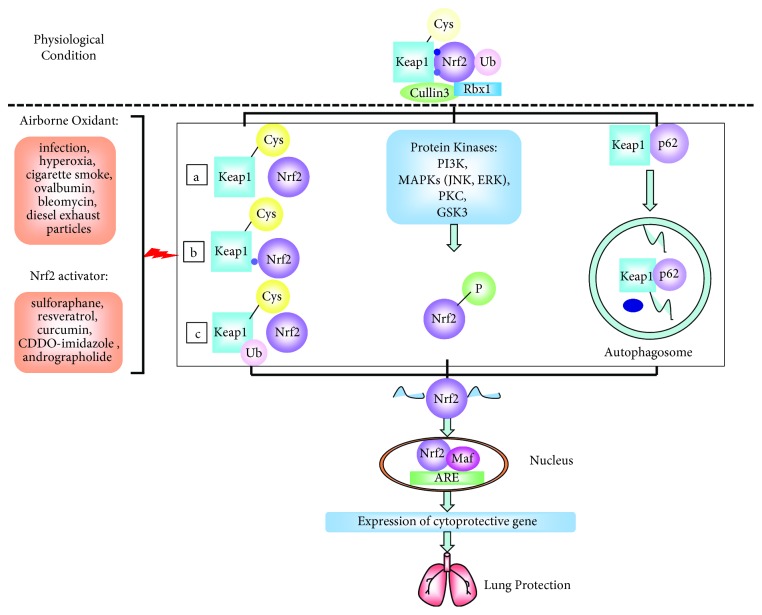
The mechanism of Nrf2 activation and Nrf2-mediated antioxidant responses in the lungs. Under unstressed conditions, Keap1 protein traps Nrf2 in the cytoplasm and targets this protein for the Cul3-Rbx1 ubiquitination system, which promotes the proteolysis of Nrf2 by the 26S proteasome. Diverse oxidative insults or pharmacological Nrf2 activators impair the ability of Keap1 to target Nrf2 for ubiquitination and degradation, promote newly synthesized Nrf2 to translocate to nucleus, and induce ARE-driven cytoprotective gene expression. Several accepted mechanisms include the modification of cysteine in Keap1: (a) Keap1 dissociation, (b) Keap1 hinge and latch, and (c) Keap1 ubiquitination. (2) There are also Keap1-independent pathways, among which protein kinases including PI3K, MAPKs, PKC, and GSK-3 play an essential role in phosphorylation of Nrf2 to increase its stability and transactivation activity. (3) Moreover, a noncanonical pathway induces Nrf2 activation by autophagy, which declares that p62 competes with Nrf2 for Keap1 binding, sequesters Keap1 into the autophagosome, and promotes its degradation. After de novo synthesized Nrf2 translocates to the nucleus, it heterodimerizes with small Maf then binds to ARE in the regulatory regions of Nrf2 target genes to induce their expression, which confer protective effects to various pulmonary diseases including BPD, respiratory infection, ARDS, COPD, asthma, IPF, and lung cancer.

The functional process occurs when de novo synthesized Nrf2 translocates to the nucleus, after which Nrf2 heterodimerizes with small Maf or Jun proteins and then binds to ARE in the regulatory regions of Nrf2 target genes and either upregulates or inhibits target genes. Previous studies have shown that in the protection of the respiratory system by Nrf2, Nrf2-targeted genes are essential effectors that are identified by microarray analyses and bioinformatic studies. The Nrf2/ARE pathway regulates more than 500 genes, including genes that regulate oxidative stress (HO-1, GCLM, and GCLC), inflammation (TGF-*β* and NF-*κ*B), xenobiotic metabolism and excretion (NQO1, AKR1C1, and MRP1), apoptosis (Bcl-2 and BclxL), and autophagy (p62) [12, 23]. Therefore, Nrf2-downstream target genes have diverse functions, including antioxidation, anti-inflammation, xenobiotic metabolism, detoxification, and cell growth regulation [25, 34, 35]. In particular, cytoprotective proteins such as HO-1, GCLM, GCLC, and NQO1 catalyze diverse detoxification reactions, converting harmful substances to hydrophilic metabolites, and are not consumed during their actions [36] (Figure 1). Moreover, the protective effects of Nrf2 are more likely to be exerted when its activation is tightly controlled. A recent study showed that once the redox homeostasis is restored or the compounds are metabolized and eliminated, signal termination will occur, and Keap1 enters the nucleus, binds to Nrf2, and then brings it back to the cytosol for degradation [37].

Accordingly, previous studies have shown that in tissues where routine antioxidation and detoxification processes occur, such as the lungs, liver, and kidneys, Nrf2 is relatively abundant. The expression level of Nrf2 is highly correlated with the susceptibility, severity, and recovery of airway disorders. Data from Nrf2-knockout mice identify the protective effects of Nrf2 on airway disorders: compared to wild-type mice, Nrf2-knockout mice have enhanced lung inflammation, epithelial cell injury, and increased sensitivity to cigarette smoke, elastase, ovalbumin, bleomycin, and other stimuli [38–41]. These findings motivate researchers to discover potential Nrf2 activators.

## 3. Protective Role of Nrf2 and Its Activators in Respiratory Diseases

As described above, the respiratory system is directly exposed to inhaled oxidants, and this oxidative burden makes this system more vulnerable to oxidant stress, which has proven to be associated with the pathogenesis of diverse respiratory diseases. Currently, the protective roles of Nrf2 signaling in respiratory disorders have been identified with the application of Nrf2 knockout mice, including three different genetic backgrounds (ICR, C57BL/6J, and Balbc/J) and lung-specific conditional knockout mice [1]. For instance, the deletion of Nrf2 is associated with more severe insults that are caused by oxidative, inflammatory, or carcinogenesis factors such as infection, hyperoxia, cigarette, ovalbumin, bleomycin, butylated hydroxytoluene, and diesel exhaust particles. Conversely, the activation of Nrf2 exerts protective effects on these lung disorders. Therefore, many studies focus on the benefits of Nrf2 inducers, including natural products, in the therapeutic intervention for oxidant-associated lung diseases [42]. In the present review, we discuss the involvement of Nrf2 in the pathogenesis of airway disorders and the protective role of diverse Nrf2 inducers in different airway disorders. Furthermore, the effects of major Nrf2 activators on different respiratory diseases are summarized in form of table (Table 1).

### 3.1. Bronchopulmonary Dysplasia (BPD)

Bronchopulmonary dysplasia (BPD) is a chronic respiratory disease that usually occurs in premature infants with very low birth weight. BPD is mainly characterized by a failure in alveolarization, which results in impaired alveolar growth and vascular development, pulmonary inflammation, and abnormal lung function [43, 44]. Furthermore, BPD not only results in significant mortality in the perinatal period but also contributes to long-term sequelae in adolescents or early adulthood with clinically significant respiratory symptoms [45].

Although the pathogenesis of BPD has not been fully understood, the lung injury in BPD can be divided into two groups described as “new” BPD and “old” BPD. The former refers to early developmental arrest by prenatal exposure or genetic factors, while the latter develops into surfactant-deficient premature infants who receive respiratory support. Further studies elucidate that important trigger events for BPD may include oxidative damage and inflammation. Particularly, as human infants undergo critical postnatal alveolar growth, therapeutically administered oxygen to improve the oxygenation of premature infants is a major risk factor, which may be associated with the induction of p21 and p53 cell cycle regulatory genes [46]. For oxidative disorders in adults, Nrf2 activation has been extensively proven to be beneficial; however, the investigation of its effects on neonatal diseases is still a novel area. As evidenced by recent studies, during the process of saccular lung maturation, Nrf2 may modulate diverse genes implicated in maintaining redox balance, organ development and lung morphogenesis, and cell growth and death as well as immunity. Indeed, previous studies indicate the effects of Nrf2 on molecular processes of alveolarization and lung diseases of premature births: deficiency in Nrf2 and exposure to hyperoxia in newborn mice increases mortality and the severity of alveolar growth inhibition, and transcriptome analysis of immature lung tissue suggests that the protection against O_2_ toxicity of Nrf2 may be mediated by its regulation of the cell cycle, metabolism processes, cell–cell interactions, and redox homeostasis [47, 48].

These findings may elucidate a possible beneficial role for Nrf2 activators in the BPD of preterm infants with respect to both lung development and hyperoxia-mediated lung injury, and several studies have attempted to unveil such potential. For instance, aurothioglucose (ATG), a TrxR1 inhibitor, inhibits thioredoxin reductase-1 (TrxR1) activity and activates the Nrf2 pathway in the lungs of newborn C3H/HeN mice exposed to hyperoxia, attenuating the decrease in body weight and alterations in alveolar development. However, the effects on alveolarization and sustained Nrf2 activation are not observed in the lungs of newborn C57BL/6 mice under the same conditions [49, 50]. In a newborn rat model of BPD, the Nrf2 activator curcumin can attenuate hyperoxic lung injury, and the protective role is considered to be at least partially related to the activation of Nrf2; unfortunately, whether the Nrf2 pathway is activated was not studied [51, 52]. Another study shows that the in utero administration of a well-recognized Nrf2 inducer, sulforaphane, can inhibit hyperoxia-induced lung inflammation in neonatal mice, but the inhibition of alveolar growth is not improved in this model of BPD [53]. Such results are provoking, and whether Nrf2 induction is beneficial under the current conditions in BPD may be controversial, and studies on the long-term effects of in utero Nrf2 inducers on alveolar growth, inflammation, and survival are needed. In consideration of these discoveries, Nrf2 activation may be a novel strategy in preventing or modulating the severity of BPD, as well as helping to improve the outcomes of the disease. However, caution should be taken when evaluating the effects of Nrf2 inducers in BPD before clinical use because the results from previous studies are still limited and controversial in some respects, such as the mode of administration, the delivery time, and even the species.

### 3.2. Respiratory Infections

As the respiratory tract directly contacts various pathogens from internal and external environments, the associated infections are highly prevalent and variable. Thus, there is still an unmet need for new therapeutic methods for these diseases, especially for infections with viruses. Recent studies indicate that during the process of respiratory infections, the excessive ROS produced by phagocytic cells usually causes an imbalance between oxidants and antioxidants, which may contribute to the pathogenesis of infection-related respiratory diseases. As an essential fighter against oxidative stress, Nrf2 is also considered to play a protective role in antiviral and antibacterial processes in several common infections of the respiratory tract.

#### 3.2.1. Respiratory Syncytial Virus (RSV) Infection

Respiratory syncytial virus (RSV) is not only a leading cause of acute respiratory tract infections in infants and children but also an essential factor for substantial respiratory morbidity and mortality in the elderly [54, 55]. However, the details regarding the pathogenesis of RSV infection have not been clarified, and effective vaccines or treatments are still not available. In recent studies, researchers have discovered that rapidly generated ROS during RSV infection are involved in lung inflammatory and oxidative damage in this clinical disease [56]. Accordingly, treatment with antioxidants can ameliorate RSV-induced pulmonary inflammation in a mouse model of RSV infection [57]. While examining the antioxidant activity of Nrf2 signaling, researchers have also detected the activation of Nrf2 during RSV infection. These results show that RSV infection induces a clear decrease in Nrf2 levels and airway antioxidant enzymes in mouse lungs as well as in child nasopharyngeal secretions, which may be due to RSV-induced Nrf2 degradation [58–61]. RSV infection promotes Nrf2 ubiquitination and degradation via the proteasomal pathway, which may be plausible because the proteasomal inhibitors MG132 and lactacystin can restore RSV-reduced Nrf2 activity. More importantly, phenotypes of RSV infection are more severe in Nrf2-deficient mice than in wild-type mice, which are reflected in more severe bronchopulmonary inflammation and epithelial injury, as well as attenuated viral clearance [62]. Studies show that the more severe airway inflammation in Nrf2^−/−^ mice induced by RSV is likely due to the enhanced concurrent activation of AP-1 and NF-*κ*B. Additionally, Nrf2 deficiency makes mice more “Th1-like” with lower GSH and IFN-*γ* levels, which may impair virus clearance. Therefore, the Nrf2 pathway may be targeted for protection against RSV-induced lung injury.

In recent years, several Nrf2 inducers have been found to display beneficial effects on RSV infection. The potent Nrf2 inducer sulforaphane pretreatment significantly limits lung RSV replication and acute inflammation in wild-type but not Nrf2-deficient mice [62]. Butylated hydroxyanisole (BHA), a compound that is also known to induce the Nrf2 pathway, can accelerate viral clearance and ameliorate RSV-induced lung inflammation in mice, which indicates that the anti-RSV activity of BHA may be derived from Nrf2 activation [57, 63]. Furthermore, a phosphodiesterase 4 (PDE4) inhibitor, roflumilast N-oxide (RNO), can reverse RSV-induced Nrf2 loss, and such effects may be associated with its inhibition of cilia activity, mucin production, and inflammatory mediators in RSV infection in well-differentiated normal human bronchial epithelial cells (WD-HBE) [64]. Similarly, the Nrf2 inducer tert-butylhydroquinone (tBHQ) can also rescue the decrease in Nrf2 activation induced by RSV [61].

#### 3.2.2. Influenza A Virus (IAV) Infection

Influenza A virus (IAV) infection ranging from upper respiratory infection to pneumonia has troubled individuals for a long time [65, 66]. Although some antiviral drugs, such as neuraminidase (NA) inhibitors, have been developed and applied, IAV infection is still a substantial threat to human health with significant morbidity and mortality due to its virus variability and resistant viral strains, including H1N1, H5N1, and H7N9 [67–69]. Thus, it is still a critical challenge to develop novel anti-influenza drugs to control influenza epidemics and pandemics in the future. In recent decades, studies have revealed that influenza viruses can induce oxidative stress, cytotoxicity, apoptosis, and inflammation in the respiratory system [70, 71]. Consistently, antioxidant compounds protect against injury induced by IAV via inhibiting virus replication and immune clearance and diminishing inflammatory mediator production [72]. Furthermore, the antioxidant pathway controlled by Nrf2 has been proven to be central in lung antioxidant defense against inflammation and injury induced by influenza virus both in human nasal epithelial cells and in mice [73, 74]. Studies have also shown that during IAV infection, Nrf2 protects human alveolar epithelial cells from the cytopathic effects induced by oxidative stress in an interferon-independent manner. The knockdown of Nrf2 makes ATI-like cells and ATII cells more vulnerable to injury, while the overexpression of Nrf2 decreases virus replication and related nucleoprotein expression [75]. However, cells from other vulnerable populations, including smokers and patients with COPD, will be needed in future studies.

To date, several Nrf2 inducers have been examined for their protective role in IAV infection. It has been recently reported that the Nrf2 inducer epigallocatechin gallate decreases influenza A/Bangkok/1/79 virus entry and replication in nasal epithelial cells; however, the inhibitory effect on replication is blocked when Nrf2 is knocked down [73], while curcumin, a classical Nrf2 inducer, not only inactivates IAV and suppresses IAV adsorption directly but also induces Nrf2 activation and inhibits IAV-induced oxidative stress, inflammation, and even IAV replication [76]. Similarly, the Nrf2 inducer sulforaphane and emodin can also lead to the significant inhibition of IAV-induced oxidative stress/inflammation and viral replication [73, 77]. Other reported compounds that may exert anti-influenza effects through the activation of Nrf2 also include bakuchiol and the rupestonic acid derivative, YZH-106 [67, 78].

#### 3.2.3. Bacterial Infections

Recently, Nrf2 has been reported to have a beneficial role in infection with diverse bacteria, and the application of Nrf2 inducers may represent a novel treatment for respiratory tract bacterial infections, although related studies are limited.

Streptococcus is an important bacterium that colonizes the upper respiratory airways. This bacterium is not only the most common cause of community-acquired pneumonia but also an essential risk factor for several life-threatening diseases, including sepsis [79]. Previous studies have shown that *S. pneumoniae* can release several bacterial components to cause an enhanced oxidant burden on the lung epithelial surface, while the specific Nrf2 inducer resveratrol can ameliorate pneumococcal-induced oxidative stress in the airway epithelium [80]. Similarly, Nrf2 can also effectively attenuate mouse lung injury and the mortality rate induced by *Staphylococcus aureus* and lung inflammation in *Haemophilus influenzae* infections [81, 82]. Furthermore, the preactivation of Nrf2 by high mobility group nucleosomal binding protein 2 (HMGN2) can attenuate the oxidative stress induced by *Pseudomonas aeruginosa* (PA) infection and inhibit the internalization of the bacteria in A549 cells [83]. Regrettably, there still lacks evidence for the application of Nrf2 activators in these infections.

Moreover, tuberculosis (TB) is a typical example of a specific infection and the top infectious disease killer worldwide. TB mainly refers to infections with *Mycobacterium tuberculosis*, especially in the respiratory tract [84, 85]. Recently, the role of oxidative stress and Nrf2 in TB infection has gained increasing attention. Oxidative stress has been proven to exist in the guinea pig model of tuberculosis along with the loss of Nrf2 activation, and the administration of NAC, an ROS scavenger and Nrf2 activator, significantly decreases bacterial burden and lung injury in TB infection, indicating a possible protective role for Nrf2 [86].

### 3.3. Acute Respiratory Distress Syndrome (ARDS)

Acute respiratory distress syndrome (ARDS) refers to the severe clinical condition of dyspnea, refractory hypoxemia, and noncardiogenic pulmonary edema and affects millions of people throughout the world. According to existing statistics, the etiology of ARDS is fairly complex, varying from severe pneumonia, sepsis, and major trauma to massive transfusion, and severe pneumonia or sepsis may be the major cause [87]. Nevertheless, the typical pathological changes in ARDS still have similar characteristics, including acute diffuse lung inflammation and the disruption of the epithelial-vascular barrier caused by the damage of epithelial and endothelial integrity [88]. These changes cooperatively result in the destruction of lung structure and function, mainly shown as impaired lung compliance and gas exchange [89]. In spite of the improvement in supportive care today, unfortunately, ARDS is still responsible for significant morbidity and mortality, owing to its elusive therapeutic methods.

Although the mechanisms responsible for the pathogenesis of ARDS have not been fully understood, there is already evidence indicating the involvement of two essential and interactional factors, oxidative stress and inflammation. The overproduction of ROS or RNS, infiltration of inflammatory cells, and synthesis of inflammatory mediators have been proven to inflict severe lung damage. Recently, increasing evidence has demonstrated the importance of Nrf2 activation in coping with oxidative stress and inflammation in ARDS. A previous study showed that Nrf2 may act as a candidate gene of ARDS susceptibility for humans, as over 500 single-nucleotide polymorphisms (SNPs) of Nrf2 have been identified to date, and the risk of ARDS after severe trauma has increased in people with a functional NRF2 SNP in European and African American individuals [90]. For experiments conducted with animals, hyperoxia or LPS-induced ARDS are perhaps two of the most well-studied models that benefit from Nrf2 activation, and Nrf2-deficient mice are extensively used in studies focused on the beneficial role of Nrf2 in ARDS. In the hyperoxia-induced rodent model of ARDS, Nrf2 is supposed to be a susceptibility gene because functional SNPs have been found in hyperoxia-susceptible C57BL/6J (B6) and hyperoxia-resistant C3H/HeJ (C3) mice, and the B6C3F2 progeny is reported to have a hyperoxia-susceptibility promoter SNP [91, 92]. Furthermore, compared to wild-type mice, Nrf2-deficient mice are more likely to develop ARDS with enhanced lung hyperpermeability, epithelium injury, and inflammation under the stimulation of hyperoxia and butylated hydroxytoluene [93, 94]. A recent study also showed that the specific deletion of Nrf2 in the airway epithelium (Clara cell) in a hyperoxia-induced ARDS model is sufficient to promote the full development of acute lung injury and delays the subsequent resolution of inappropriate lung inflammation and epithelial sloughing [95]. For the LPS-induced ARDS model, in addition to increasing susceptibility and severity in Nrf2-deficient mice, it is also intriguing to find that Nrf2 may provide protective effects on LPS-induced ARDS via its promotion of M2 polarization in macrophages in a recent study adopting the Nrf2 activator tert-butylhydroquinone (tBHQ) and Nrf2 siRNA [96].

Therefore, in recent years, numerous investigations have focused on the protection against ARDS by Nrf2 activators, especially in hyperoxia- or LPS-induced ARDS models. The oleanane triterpenoid CDDO-imidazole (CDDO-Im) is reported to activate Nrf2/ARE signaling by breaking the interactions between Keap1 and Nrf2 in the cytosol and confer protective effects such as the inhibition of pulmonary hemorrhage, proteinaceous edema, and inflammatory cell infiltration on hyperoxia-induced ARDS. However, these protective effects are almost abolished in Nrf2-deficient mice [97]. Moreover, this compound also dampened aspiration-induced ARDS in another experiment. Importantly, its analogs, CDDO-methyl esters, have already been used in clinical trials for cancer treatment (http://clinicaltrials.gov), which brings the hope of the clinical application of CDDO-Im. Similarly, vitexin and aucubin both mitigate inflammatory and oxidative injury along with the suppression of inflammatory signaling, such as NLRP3/NF-*κ*B, and the induction of Nrf2 in the LPS-induced ARDS model in wild-type but not Nrf2 knockdown mice/macrophages [98, 99]. The most well-recognized Nrf2 inducer, sulforaphane, also exerts protective effects on an ARDS murine model induced by LPS via inhibiting the increase of NF-*κ*B and activating the Nrf2 pathway and is also reported to alleviate lung injury in another oleic acid-induced ARDS model [100, 101]. Indeed, a large number of compounds have been reported to act in a similar manner that activates Nrf2 signaling in an LPS-challenged ARDS model, such as resveratrol, alpha-lipoic acid, ethyl gallate, cordycepin, and syringin [102–107]. More intriguingly, another potent Nrf2 activator, tBHQ, protects against LPS-induced lung injury in an ARDS mouse model by promoting the polarization of M2 macrophages, suppressing the polarization of M1 macrophages and modulating the balance between proinflammatory and anti-inflammatory factors [96]. Furthermore, the transfection of Nrf2 is also able to reinforce the efficacy of human amniotic mesenchymal stem cells (hAMSCs) to inhibit inflammation, fibrosis, and lung injury and promote hAMSCs to differentiate into type II alveolar epithelial (AT II) cells with enhanced activity of Nrf2 in an LPS-induced mouse ARDS model [108].

### 3.4. Chronic Obstructive Pulmonary Disease (COPD)

Chronic obstructive pulmonary disease (COPD), a common chronic pulmonary disease characterized by irreversible airflow limitation, is projected to emerge as the third most prevalent cause of death by 2020 [109]. The pathological basis of COPD is associated with small airway obstruction and tissue remodeling, which may result from abnormal inflammation and lung parenchyma destruction in response to diverse stimuli, including genetic/environmental risk factors and their complicated interactions [110, 111]. Moreover, in recent decades, oxidative stress has also been recognized as an important predisposing factor that accounts for the pathogenesis of COPD, and cigarette smoking (CS) containing rich oxidants and other detrimental substances has long been regarded as a dominant environmental risk factor for COPD [112].

Exposure to cigarette smoke can lead to obvious oxidative stress, inflammation, and alveolar cell apoptosis. In healthy smokers, to cope with such high oxidant burden, there is a significant increase in numerous antioxidant defenses, among which Nrf2 is largely relied on. This reliance may be evidenced by the transient Nrf2 expression induced by CS in human airway cells. However, decreased levels of Nrf2 and its stabilizer DJ1 (PARK7) in lung tissues of COPD patients have been observed, and multiple human studies have demonstrated that the NRF2–KEAP1–BACH1 equilibrium in lung and alveolar macrophages is lowered in the population of aged smokers and COPD patients. Moreover, a cohort study on the relationship between polymorphisms of the Nrf2 gene and limitations of airflow in smokers also indicates that impaired Nrf2 may contribute to the development of COPD owing to excessive oxidant burden and apoptosis in the lungs [113]. The severity of COPD and the occurrence of respiratory failure may also be associated with the haplotype of the Nrf2 gene promoter, which affects its activity [114]. Consistent with these findings, Nrf2-deficient mice are more susceptible to cigarette smoke exposure and develop more severe lung emphysema and apoptosis, and the activity of antioxidant enzymes is repressed [40]. Additionally, the relationship between Nrf2 and COPD has also been addressed in vitro: Nrf2 knockdown increases 10% cigarette smoke exposure- (CSE-) induced apoptosis, while Nrf2 overexpression protects cells from apoptosis induced by CSE. Notably, the deletion of Keap1 and subsequent activation of Nrf2 signaling in Clara cells not only protects cells against oxidative stress ex vivo and in human epithelial cells but also suppresses oxidative stress and CS-induced inflammation in vivo [115]. Furthermore, Nrf2 has an influence on the infection-related acute exacerbations and therapeutic responses to corticosteroids in COPD. During COPD, oxidative stress has been reported to disrupt innate immune defenses and amplify inflammation. On the one hand, Nrf2 deletion impairs the clearance of respiratory infections via its effects on scavenger receptor macrophage receptor with collagenous structure (MARCO). On the other hand, a reduction in HDAC2 activity may lead to increased inflammation and corticosteroid resistance. A recent study has already shown that CS-exposed and LPS-induced Nrf2^−/−^ mice display augmented lung inflammation that cannot be alleviated by steroids and revealed that deficits in Nrf2 may play an essential role in steroid resistance via HDAC2 repression: the recruitment of HDAC2 is important in mediating the anti-inflammatory activities of glucocorticoids by its interaction with promoters of proinflammatory genes, while Nrf2 deficiency may significantly reduce histone deacetylase 2 (HDAC2) level and deacetylase activity [116].

Therefore, novel therapies, such as Nrf2 activators, may show promise for therapy in COPD patients. Several Nrf2 activators have the potential to protect against exposure to cigarette smoke. For example, upon chronic CS exposure, CDDO-Im induces a more significant upregulation of Nrf2 and its target genes, mitigating CS-induced lung oxidative stress, tissue destruction, and even pulmonary hypertension in wild-type mice; however, these protective effects are not obviously observed in Nrf2-deficient mice. Additionally, it is interesting that CDDO-Im shows no inhibitory effect on CS-induced inflammation, despite its prevention of emphysema development, which may suggest that the inhibition of oxidative stress is enough to interrupt the development of emphysema [117]. Similarly, the WNT activator LiCl suppresses emphysematous changes and the lung inflammation induced by elastase or CSE, respectively, in WT mice along with the upregulation of Nrf2 signaling; however, such protective effects are almost abolished in elastase-challenged Nrf2-deficient mice. Additionally, the protective effects of the AMPK activator metformin on CSE-stimulated increases in inflammatory markers in Nrf2-deficient NHBE cells are not obviously observed [118]. Moreover, two well-known Nrf2 activators, sulforaphane and andrographolide, not only protect against CS/CSE-induced injury but also act in controlling infections that exacerbate COPD. In a recent study, sulforaphane was reported to counteract the oxidative injury induced by CSE with the activation of Nrf2 signaling in a rat alveolar epithelial cell line [119]. Furthermore, sulforaphane can augment the phagocytosis of bacteria (such as PA and NTHI isolated from COPD patients) by alveolar macrophages from COPD patients, but the ability is absent in Nrf2 siRNA-transfected macrophages, and similar results are obtained in wild-type and Nrf2 knockout mice. The study further shows that the Nrf2-dependent bacterial clearance by sulforaphane may be associated with Nrf2-mediated regulation of scavenger receptor MARCO but not dependent on the antioxidant glutathione [120]. However, although these findings suggest that sulforaphane protects against COPD and COPD exacerbation, the administration of sulforaphane to COPD patients unfortunately failed to induce Nrf2 target gene expression or affect the levels of other antioxidants or inflammation markers in a randomized, double-blind, placebo controlled trial in the US [121]. Another typical compound for COPD treatment, andrographolide, is a kind of lactone extracted from *Andrographis paniculata*. Recent studies reveal that andrographolide has the ability to protect the lungs from oxidative injury caused by cigarette smoke and suppress nontypeable *Haemophilus influenza*- (NTHi-) increased inflammatory and oxidative lung injury in a CS-predisposed mouse model that imitates COPD exacerbation, while the mechanism of action may be attained by the induction of Nrf2-mediated cytoprotective responses [122, 123]. Intriguingly, this compound has already been applied as a drug component in some areas of China. In addition, influenza virus (FluV) is important in the acute exacerbations of COPD, and a recent study found that CS-exposed Nrf2^−/−^ mice showed increased mortality and lung damage of increased severity after FluV infection [74]. Therefore, Nrf2 inducers that are effective in FluV infection, such as curcumin, may also provide protection to certain acute exacerbations of COPD. Indeed, there are many other compounds that have been reported to protect against lung tissue or epithelial injury induced by CS/CSE via the modulation of Nrf2, such as resveratrol, ursolic acid, the vitamin E isoform *γ*-tocotrienol, and aspirin-triggered resolvin D1 (AT-RvD1) [124–127].

### 3.5. Asthma

Asthma is a complex respiratory disorder characterized by chronic airway inflammation, airway hyperreactivity (AHR), and extensive and polytropic reversible airway obstruction [128]. Supported by previous studies, oxidative stress is highly involved in the pathogenesis of human asthma, and the oxidant burden plays a pivotal role in airway inflammation, AHR, and even insensitivity to steroids [129, 130]. As reported, patients with asthma may have more problems coping with oxidant burden than healthy people, which may be intimately related to impaired Nrf2 activity. Recent studies have also shown that Nrf2-driven GST is a possible marker for asthma susceptibility in humans: the homozygous GSTM1-null genotype increases the risk for asthma, but homozygous GSTP1 expression can protect against asthma [131, 132]. In mice, it has also been reported that Nrf2 deficiency significantly enhances ovalbumin or diesel exhaust particle- (DEP-) driven oxidative stress, airway inflammation, and AHR in an asthma model [41, 133]. In the OVA-challenged asthma model, the disruption of Nrf2 not only leads to increased levels of eosinophils, particularly in BALF and lung tissues, in Nrf2^−/−^ mice but also causes higher levels of neutrophils, which may be responsible for airway remodeling in severe asthma. In turn, eosinophils and neutrophils generate more ROS to cause damage to the lung. In addition, the disruption of Nrf2 may also be associated with more obvious AHR, goblet cell hyperplasia, epithelial cell apoptosis, and an elevated level of Th2 cytokines in this model. In the DEP-challenged asthma model, Nrf2^−/−^ mice also display increased eosinophils, AHR, IL-12, IL-13, and thymus and activation-regulated chemokines (TARC) in BALF. Moreover, when dendritic cells (DCs) from both Nrf2-deficient and wild-type cells were exposed to particulate matter (PM), Nrf2 successfully restrains the production of a proallergic phenotype via the inhibition of oxidative stress and Th2-directed proallergic immunity regulated by DCs [134]. During this process, the constitutive immune-polarizing cytokine milieu due to Nrf2 deficiency in DC plays an essential role in the augmenting promoting effect of PM on allergic sensitization. Taken together, Nrf2-mediated antioxidant responses can serve as an important determinant of susceptibility to asthma.

In recent decades, the protective role of Nrf2 inducers in asthma has been extensively investigated, and ovalbumin is used as a classical asthma inducer. Until recently, different studies have examined the protection of sulforaphane against asthma. Sulforaphane administration can suppress ovalbumin-induced allergic airway inflammation in mice, and a recent study further investigated the protective effects of sulforaphane in asthmatics. The results reveal that Nrf2 signaling may play an essential role in individuals whose bronchoprotective responses against methacholine (MCh) are improved by sulforaphane [135, 136]. Moreover, in a Cl_2_-induced murine asthma model defined as irritant-induced asthma (IIA) and DEP-stimulated airway epithelial cells, sulforaphane administration with augmented Nrf2 activity inhibited airway inflammation [137, 138]. Notably, sulforaphane administration in healthy human subjects fails to increase Nrf2 and its target antioxidant gene expression but protects against neutrophilic airway inflammation induced by ozone [139]. Therefore, whether sulforaphane can be used clinically still needs more evidence from studies on different populations and allergens. Similarly, another Nrf2 inducer, sappanone A (SA), can protect against allergic airway inflammation induced by OVA, inhibiting the increase of inflammatory cells, cytokines, and OVA-specific IgE in BALF and restoring the level of IFN-*γ*, and the mechanism of action may derive from Nrf2-regulated Th1/Th2 balance [140]. The well-known antioxidant vitamin E (*α*-tocopherol) is also reported to protect against IgE-induced asthma by reversing the impairment of Nrf2 activity in alveolar macrophages in vivo and alleviating asthma exacerbation stimulated by ozone in the OVA-induced murine model via the Nrf2 pathway, although its effect is absent on OVA-induced asthma symptoms [141, 142]. Moreover, compounds such as vitamin D3, forsythiaside A (FSA), and mainstream anti-malarial drug artesunate all have similar effects on the amelioration of airway inflammation and AHR in OVA-induced asthma through activating the Nrf2/HO-1 signaling pathway [143–145].

### 3.6. Idiopathic Pulmonary Fibrosis (IPF)

Idiopathic pulmonary fibrosis (IPF), an important representative of interstitial lung disease, is described as a chronic progressive lung disease with fibroproliferation and excessive extracellular matrix (ECM) deposition, which ultimately results in irreversible lung interstitial fibrosis and respiratory failure [146]. Although great efforts have been made to combat this disease, unfortunately, no treatment is reported to have actual benefits to improve the survival rate. Like many other chronic diseases, the pathogenesis of IPF is still unknown; however, oxidative stress is closely implicated according to previous studies, in which increased oxidative burden has already been observed in IPF patients [147, 148]. Oxidative stress-driven insults of lung tissue may come from different dimensions. For example, excessive ROS results in DNA damage and apoptosis in lung epithelial cells and drives IPF progression via triggering the activation and release of TGF-*β*1, which can accelerate ROS generation, existing inflammation, and lung scarring as well as suppress antioxidant gene expression by mediating the interaction of Smad3-ATF3 with Nrf2. Moreover, ROS play an essential role in myofibroblastic differentiation, which is intimately involved in the pathogenesis of IPF. Therefore, it is not surprising that the dysregulation of Nrf2, a master regulator of oxidative stress, is reported to contribute greatly to pulmonary fibrosis [149]. In previous studies, Nrf2-deficient mice are more susceptible to the inducer of IPF-like lung fibrosis and bleomycin than wild-type mice, and these deficient mice more obviously display lung inflammation and fibrogenesis, along with increased fibrosis indices and decreased antioxidant response [38]. In vitro, Nrf2 siRNA leads to not only augmented oxidant burden but also increased myofibroblastic differentiation, whereas the knockdown of Keap1 induces the opposite effects. Nevertheless, evidence in humans for similar conclusions still remains to be explored, although augmented Nrf2 expression accompanied by oxidant markers is found in lung tissues from IPF patients, especially when the major inflammatory response and the oxidant nature in the common model induced by bleomycin still contrast with the histological features of human IPF.

Several Nrf2 activators have been studied to protect against pulmonary fibrosis. Pirfenidone (PFD) is a current approved drug for the therapy of IPF, and its antifibrosis activity in transforming growth factor-*β*- (TGF-*β*-) stimulated fibroblasts and bleomycin-challenged murine models is associated with the restoration of Nrf2/Bach1 equilibrium through Bach1 inhibition and Nrf2 activation [150]. The classical Nrf2 activator sulforaphane also shows antifibrosis effects on IPF fibroblasts in vitro by reversing the hallmarks of myofibroblastic differentiation (such as the increase of *α*-SMA, collagen I, fibroblast proliferation, migration, and contraction), even under TGF-*β* stimulation, and the antifibrosis activity is dependent on the restoration of redox balance by Nrf2 activation. However, studies have reported that sulforaphane cannot protect against bleomycin-induced lung fibrosis in mice, which may be related to the fact that this model does not address the effects of Nrf2 activators in lung fibrosis due to the absence of Nrf2 activation in mouse lung fibroblasts [151]. Another Nrf2 inducer, emodin, similarly suppressed BLM-induced fibrotic lung injuries in rats via the inhibition of collagen overproduction, epithelial-mesenchymal transition (EMT), and TGF-*β* and p-Smad expression. In addition, emodin can reverse recombinant TGF-*β*1-stimulated EMT-like shifts in alveolar epithelial-cultured cells [152]. Additionally, quercetin significantly induces antioxidant defense via Nrf2 activation and suppresses inflammation in bleomycin-challenged BEAS-2B cells, and its inhibitory effect on TGF-*β*-induced fibrosis in fibroblasts is also at least partly mediated by HO-1, a main downstream target of Nrf2 [153, 154]. Apart from the above compounds, the antifibrotic effects of berberine and epigallocatechin-3-gallate (EGCG) on bleomycin-induced pulmonary fibrosis in a murine model may also be at least partly mediated by the activation of Nrf2 signaling, accompanied by the inhibition of inflammation and other biological events [149, 155].

### 3.7. Lung Cancer

Lung cancer is reported to be the leading cause of cancer-related mortality worldwide [156]. In all types of lung cancer, non-small-cell lung carcinoma (NSCLC) is the most common and has been extensively studied on its different subtypes, while small-cell lung carcinoma (SCLC) only accounts for approximately 20% of lung cancer [157, 158]. As this disease is prone to be diagnosed late and the response to existing drugs is poor, patients with lung cancer always have poor prognoses. Therefore, novel possible drugs to prevent this disease and improve the outcomes are still in urgent need.

To decrease the incidence of cancer, people attempt to apply chemicals to detoxify or remove carcinogens [159]. Among these potential chemicals, Nrf2 inducers have been proven to have special advantages as chemopreventive agents [160]. However, the effects of Nrf2 on cancer pathogenesis are still controversial in the lungs [161]. In addition to cytoprotective functions, Nrf2 and its targeted genes also take part in several oncogenic signaling pathways, such as PI3K and K-ras, and connect with other transcription factors, structural proteins and epigenetic enzymes involved in the pathogenesis of cancer [162]. Nrf2 indeed displays opposing biological activities during carcinogenesis. Therefore, numerous studies have attempted to reveal the precise role of Nrf2 in lung cancer and have gained certain possible points. In a vinyl carbamate/urethane-induced and Kras^G12D^-driven genetic lung cancer mouse model, Nrf2 is able to inhibit lung cancer initiation in a vinyl carbamate/urethane-induced model but promotes existing tumor progression in either model [163, 164]. Another study showed abnormal immunity associated with impaired Nrf2 in vinyl carbamate-induced lung cancer. Upon vinyl carbamate challenge, the lungs and tumors in Nrf2-deficient mice display the increased infiltration of immune cells that promote the development of tumors and upregulated gene expression that responds to the immune response. Data in patients with lung cancer also provide support for the importance of such findings [165]. The possible protective effects of Nrf2 on certain stages of lung cancer have also been observed in several other studies. For instance, coal tar pitch- (CTP-) induced malignant BEAS-2B cell transformation is accelerated when Nrf2 expression is knocked down [166]. In the Lewis lung carcinoma metastasis model of mice, Nrf2 is reported to prevent carcinoma metastasis because Nrf2-deficient mice have more metastatic nodules than wild-type mice [167]. These findings indicate that determining whether Nrf2 activation is protective may depend on the stage of lung cancer. Nevertheless, although Nrf2 exerts a chemopreventive effect on certain lung cancer mouse models, related clinical evidence is still lacking. In contrast, some clinical evidence indicates that the constitutive upregulation of Nrf2 is related not only to cancer progression but also to resistance to traditional therapy and worse outcomes in NSCLC [164, 168]. According to an investigation conducted by The Cancer Genome Atlas (TCGA), changes in the Keap1/Nrf2/Cullin3 pathway exist in a third of squamous cell lung cancer, while the rate may be variable due to the number of study subjects and cancer subtypes in different studies [169]. Keap1 dysfunction due to somatic mutation/methylation and abnormal Nrf2 activation in NSCLC patients may be related to worse progress-free and overall survival. Conversely, silencing Nrf2 by siRNA in cells inhibits tumor growth and reverses chemotherapeutic resistance. Similarly, Nrf2 activation induced by mutant p53 in NSCLC mediates the resistance of cisplatin-based chemotherapy and leads to poor prognosis [170].

Considering the double effect of Nrf2 on lung cancer, the application of the Nrf2 activator in the early stages of carcinogenesis to prevent cancer may be more promising. Indeed, several well-known Nrf2 inducers have been studied in this respect. One of the most potent activators of Nrf2, sulforaphane, activates Nrf2 signaling via its impact on Keap1 and exerts suppressive effects on benzo(a)pyrene- (B(a)P-) initiated lung carcinogenesis in mice [171]. Intriguingly, another study revealed that in untransformed BEAS-2BR cells, sulforaphane activates the Nrf2 pathway and inhibits ROS production, ultimately repressing malignant cell transformation. However, in cadmium-transformed BEAS-2BR cells, sulforaphane suppresses the constitutive activation of Nrf2 and alleviates apoptosis resistance. The dual effects of sulforaphane make this compound a possible ideal agent for protection against cadmium-induced carcinogenesis, including lung carcinogenesis [172]. Curcumin, another classical Nrf2 activator, is reported to have chemopreventive efficacy in different models and be a radiotherapy/chemotherapy sensitizer or protector [173]. A recent study revealed that curcumin exerts anti-initiating effects via reducing phase I and inducing phase II enzymes in B[a]P-treated mice [174]. However, curcumin displayed limited effects in clinical trials due to its poor bioavailability [175]. To overcome such disadvantages, researchers have studied its derivatives with better bioavailability. Intriguingly, one of its major derivatives, bis[2-hydroxybenzylidene]acetone (BHBA), also significantly upregulates Nrf2 signaling, counteracts sodium arsenite- [As(III)-] induced cytotoxicity in a lung epithelial cell line and inhibits tumor formation in a vinyl carbamate-induced lung adenocarcinoma model of A/J mice [176]. Moreover, CDDO-methyl ester (CDDO-Me), a synthetic oleanolic triterpenoid similar to CDDO-Im, has been reported to significantly activate the Nrf2 pathway and reduce the number, size, and severity of tumors in vinyl carbamate-induced lung carcinogenesis in A/J mice. Additionally, CDDO-Im has semblable activity, although it seems less potent [177]. In addition, the inhalation of the Nrf2 inducer oltipraz can also suppress B(a)P-initiated lung adenocarcinoma in A/J mice [178].

## 4. Concluding Remarks

Oxidative stress has been reported to participate in the occurrence and development of various respiratory diseases, including BPD, respiratory infection, ARDS, COPD, asthma, IPF, and lung cancer. Thus, the master antioxidant transcription factor Nrf2, which is abundant in the lungs, drives diverse cellular defense pathways to counteract detrimental stimuli that are involved in the pathogenesis of these pulmonary disorders, including oxidative stress, inflammation, apoptosis, and carcinogenesis. Currently, Nrf2-deficient mice have provided an effective tool for investigating Nrf2 function in oxidative pulmonary disease models and have led to a better understanding of Nrf2 function in the pathogenesis of related pulmonary diseases. In fact, researchers have adopted three different genetic backgrounds (ICR, C57BL/6J, and Balbc/J) and lung-specific conditional knockout mice to conduct experiments to obtain insight into the protective roles of Nrf2. Therefore, in recent decades, a large number of studies have focused on the protective effects of Nrf2 activators on the above pulmonary diseases and found that certain compounds may provide new strategies to intervene or prevent oxidative airway diseases via Nrf2 induction.

## Figures and Tables

**Table 1:** The effects of major Nrf2 activators on different respiratory diseases.

Nrf2 activator	Respiratory disease	Effects	Ref.
Curcumin	BPD	Attenuates hyperoxic lung injury in newborn rats	[46, 47]
	IAV infection	Inactivates IAV and inhibits IAV-induced oxidative stress, inflammation, and IAV replication	[71]
	Lung cancer	Exerts anti-initiating effects in B(a)P-treated mice	[167]

Derivative BHBA	Lung cancer	Counteracts As(III)-induced cytoxicity in lung epithelial cells and inhibits tumor formation in vinyl carbamate-induced lung cancer	[169]

Sulforaphane	BPD	Inhibits hyperoxia-induced lung inflammation in neonatal mice	[48]
	RSV infection	Limits lung RSV replication and acute inflammation	[57]
	IAV infection	Inhibits oxidative stress and viral replication	[68]
	ARDS	Exerts protective effects on LPS and oleic acid-induced ARDS murine model	[95, 96]
	COPD	Counteracts CSE-induced oxidative injury in alveolar epithelial cells and augments bacteria phagocytosis by alveolar macrophages	[112, 113]
	Asthma	Inhibits DEPs-stimulated inflammation in airway epithelial cells, suppresses ovalbumin and Cl2-induced allergic airway inflammation in mice, and improves bronchoprotective response against MCh in asthmatics	[128–131]
	IPF	Provides antifibrosis effects in IPF fibroblasts even under TGF-*β* stimulation	[144]
	Lung cancer	Exerts suppressive effects on B(a)P-initiated lung carcinogenesis in mice and inhibits ROS production and malignant cell transformation in untransformed BEAS-2BR cells, but alleviates apoptosis resistance in cadmium-transformed BEAS-2BR cells	[164, 165]

tBHQ	RSV infection	Rescues decrease in Nrf2 activation induced by RSV	[56]
	ARDS	Protects against LPS-induced lung injury via regulating polarization of macrophages and balance of pro- or anti-inflammatory factors	[91]

Resveratrol	Pneumococcal infection	Ameliorates pneumococcal-induced oxidative stress in the airway epithelium	[75]
	ARDS	Protects against LPS-induced ARDS	[97]
	COPD	Protects against CS-induced lung injury	[118]

CDDO-Im	ARDS	Inhibits lung injury in hyperoxia and aspiration-induced ARDS	[92]
	COPD	Mitigates CS-induced lung oxidative stress, tissue destruction, emphysema development, and even pulmonary hypertension in mice	[110]
	Lung cancer	Reduces number, size, and severity of tumors in vinyl carbamate-induced lung carcinogenesis	[170]

Analogue CDDO-Me	Lung cancer	Reduces number, size, and severity of tumors more potently in vinyl carbamate-induced lung carcinogenesis	[170]

Andrographolide	COPD	Protects lung from CS-induced oxidative injury and suppresses NTHi-increased inflammatory and oxidative lung injury in a CS-predisposed mouse model that imitates COPD exacerbation	[115, 116]

Vitamin E	Asthma	Protects against IgE-induced asthma and alleviates asthma exacerbation stimulated by ozone in the OVA-induced murine model	[134, 135]

Isoform *γ*-tocotrienol	COPD	Protects against lung injury induced by CS	[120]

Emodin	IAV infection	Inhibits IAV-induced oxidative stress/inflammation and viral replication	[72]
	IPF	Suppresses BLM-induced fibrotic lung injuries in rats and reverses recombinant TGF-*β*1-stimulated EMT-like shifts in alveolar epithelial cultured cells	[145]

Quercetin	IPF	Induces antioxidant defense and suppresses inflammation in bleomycin-challenged BEAS-2B cells and inhibits TGF-*β*-induced fibrosis in fibroblasts	[146, 147]
